# Editorial: Neural Control of Energy Homeostasis and Energy Homeostasis Regulation of Brain Function

**DOI:** 10.3389/fnins.2022.872296

**Published:** 2022-04-01

**Authors:** Lionel Carneiro, Claude Knauf, Virginie Mansuy-Aubert

**Affiliations:** ^1^Department of Biological Chemistry and Pharmacology, Ohio State University, Columbus, OH, United States; ^2^Université Paul Sabatier, Toulouse III, INSERM U1220, Institut de Recherche en Santé Digestive (IRSD), CHU Purpan, Toulouse, France; ^3^International Research Project (IRP), European Lab “NeuroMicrobiota”, Toulouse, France; ^4^Department of Cell and Molecular Physiology, Stritch School of Medicine, Loyola University Chicago, Maywood, IL, United States

**Keywords:** obesity, diabetes, neurological disorders, nutrition, gut brain axis

In recent years, research related to metabolic disorders has led to numerous discoveries linking metabolic control, and age-related diseases including cognitive deficiencies. It is now established that glucose homeostasis dysregulations are risk factors for dementia, but also Alzheimer and Parkinson's diseases or even mood disorders. Thus, nutritional approaches have been proven successful on cognitive improvement in people with neurological disorders (Carneiro and Pellerin). Altogether, these observations indicate that energy homeostasis regulation is of great importance for normal brain function. Consequently, recent attention has grown in order to decipher the mechanisms lying beneath the interplay between metabolism and brain activity. For instance, a stress response involves a regulatory loop from brain to periphery that involves the hypothalamus. Interestingly, the hypothalamus is well known for its contribution to metabolic regulations such as body weight or blood glucose among others. Blood glucose is an internal parameter described as affected by stress hormones such as cortisol. Thus, the development of a new system able to regulate energy levels in hypercortisolism represents a significant advance in the management of disorders such as Cushing's disease. Hence helping to improve brain activity by lowering specific symptoms such as fatigue and disturbed sleep (Fekri Azgomi et al.).

In accordance with a brain function dependence on energetic status, Das et al. demonstrate an interplay between brain energy metabolism and cognitive dysfunction in Alzheimer's disease (AD). In their article, the authors developed a highly sensitive system to measure brain energy metabolism in early stages of AD. Such tool would be of help for an early diagnosis of the disease. Moreover, it could contribute to a better management of AD as well. In addition, their work supports a key role of brain energy metabolism on AD, and hence, supports a role for the energy balance in neurological disorders (Das et al.). In this regard, a key role of nutrient sensing in brain function in addition to energy homeostasis would not be surprising. Accordingly, the results provided by Garcia et al., studying the cell types involved in the palatability of food are of great interest. Indeed, the authors demonstrate the role of GABAergic neurons of the lateral hypothalamic area on the coding of palatability. In fact, the results provided indicate that these GABAergic neurons display activity changes in response to differences in palatability. Furthermore, the activation of these neurons stimulates food intake of food with the highest palatability (Garcia et al.). The results presented by Garcia et al., also highlight the importance of hedonism on food ingestion. By so, this study supports the role of a psychological aspect on brain control of metabolism. This role is further described in the innovative view of the phantom limbs experience applied to people following a bariatric surgery (Gautron). In his article Gautron describes how subjects undergoing a bypass surgery still experience satiation while the surgery is associated with a complete denervation. Indeed, the stimulation of mechanoreceptors of the stomach are necessary to induce satiation. Therefore, Gautron present a new hypothesis on changes in nerve distribution to preserve the satiation response. Although still hypothetic, this review supports the existence of a complex interplay between biological, bioenergetics and psychological aspects in metabolic regulations. In fact, data obtained from bariatric surgery have highlighted the key role of gut-brain axis in physiology. The gut is the first organ to sense nutrients and thus, is the first organ involved in metabolic control. The development of gut-brain axis research has also contributed to a better understanding of the link between brain function and energy homeostasis. Indeed, in addition to the nervous connections between the gut and the brain (An et al.), it is now established that products of gut metabolism are also linked with brain activity. Among these signals, SCFA and ketone bodies are the most studied. However, many other could be also involved.

Interestingly, this close relationship between brain function and energy homeostasis partly accounts for the increased risk of neurological disorders in diabetic people. On the other hand, Niu et al., present data showing behavioral side effects of the use of oxytocin for body weight management. Hence, it appears important to pursue research efforts to better understand the mechanisms involved in the relationship between cognition and metabolism. A better knowledge of this relationship would be important to address the side effects in the current therapies used. In fact, this dual effect of oxytocin in weight management and prosocial behavior could represent a promising research track for both metabolic and cognitive research fields.

The link between brain function and energy homeostasis regulating circuits within the brain is assessed in the study of Li et al. In their study the authors describe a link between binge eating and stress response. In fact, the authors showed that the reward system in BEPs rats overcame the homeostasis and stress response systems. This work supports an interplay between, stress, reward and energy homeostasis regulation. Moreover, it appears that those systems are capable of regulating each other (Li et al.). In accordance with that, the work of Perissinotti et al. showing the involvement of TRPC channel on the POMC expressing neurons depolarization supports the need to dig deeper on the neural control of metabolism. This study first demonstrates that TRPC alone can induce a POMC expressing neurons excitability. Furthermore, they also report the role of t-type channels in such POMC neurons excitability. Altogether their work brings up a putatively new protein involved in obesity development including t-type channels and KLHL1. Both proteins participate in the sensitivity to leptin of POMC expressing neurons. Thus, although POMC neurons have been identified decades ago, Perissinotti et al., studies indicate that the complete understanding of the function of these neurons are far to be completely elucidated (Perissinotti, Martínez-Hernández, and Piedras-Rentería; Perissinotti, Martínez-Hernández, He, et al.). Furthermore, the study of early developmental organization of neural circuits should also help understand the complexes interplay between different brain regions and brain functions. Thus, Lanzillo et al. provide interesting data describing the ontogeny of the BNST projections to the hypothalamus. Such work highlights the early link between stress response systems and metabolic control ones. Since the BNST is well known for mechanisms disturbed in psychiatric disorders, this suggests a putative early link between metabolism and brain disorders (Lanzillo et al.).

Altogether, these recent researches highlight the numerous gaps still existing on the understanding of neural control of energy homeostasis. Therefore, more research is needed to completely decipher the mechanisms at play. In this aspect, studies on models such as fish could help identify new mechanisms involved in metabolic control and brain function (Soengas). Eventually, one could expect to better apprehend the link between metabolism and neurological function.

Overall, the publications presented in this Research Topic support a link between brain control of energy homeostasis and brain activity. It also shows that despite extensive work made in recent years, several mechanisms are yet to be discovered. In particular, the mechanisms linking energy homeostasis and brain function are far to be clearly identified. In fact, a large number of studies indicate a clear relationship between neuronal disorders and metabolism. Therefore, metabolic studies aiming at addressing the impact on brain disorders as well as studies looking at brain disorders impact on metabolic control are still needed. Finally, such researches could help to better treat both metabolic and neuronal disorders.

## Author Contributions

All authors listed have made a substantial, direct, and intellectual contribution to the work and approved it for publication.

## Conflict of Interest

The authors declare that the research was conducted in the absence of any commercial or financial relationships that could be construed as a potential conflict of interest.

## Publisher's Note

All claims expressed in this article are solely those of the authors and do not necessarily represent those of their affiliated organizations, or those of the publisher, the editors and the reviewers. Any product that may be evaluated in this article, or claim that may be made by its manufacturer, is not guaranteed or endorsed by the publisher.

